# Otolaryngology Residents’ Perceptions of Pregnancy and Parental Leave During Training

**DOI:** 10.1017/S0022215125102594

**Published:** 2025-10

**Authors:** Amanda Walsh, Jasmine Gulati, Veranca Shah, Isabel Snee, Sarah Rapoport, Kelly Scriven

**Affiliations:** 1 Department of Otolaryngology – Head & Neck Surgery, Washington, DC USA; 2 Georgetown University School of Medicine, Washington, DC USA; 3 District of Columbia Veterans Affairs Medical Center, Washington, DC USA

**Keywords:** Medical education, Otology, Rhinology, Head and Neck Surgery, Sleep medicine

## Abstract

**Objective:**

To evaluate the experiences and perspectives of otolaryngology residents regarding current parental leave (PL) practices, incorporating insights from both male and female trainees to assess institutional policies and support mechanisms.

**Methods:**

A 43-item anonymous survey was distributed to 125 ACGME-accredited otolaryngology residency programs, yielding responses from 105 residents (response rate: 29%). Data were analyzed using descriptive statistics and thematic analysis to evaluate perceptions of PL policies, barriers to leave, and postpartum support.

**Results:**

Most respondents were female (57%) and married (77%), with an average age of 30 years. Only 26% were aware of the American Board of Otolaryngology’s 8-week PL policy. Female residents typically took 4-6 weeks of leave, while male residents took none. Concerns about program strain (29%) and lack of lactation support (60%) were significant barriers. Despite this, most respondents felt supported by co-residents and faculty.

**Conclusion:**

Otolaryngology residents reported dissatisfaction with PL policies, inadequate lactation support, and poor awareness of institutional guidelines. Addressing these issues is critical to fostering a supportive environment for residents pursuing parenthood during training.

## Introduction

The field of medicine has seen a considerable increase in the number of female students, residents, and physicians.^1^ The 2023-2024 medical school application cycle reported that women accounted for 56.6% of applicants, 55.4% of matriculants and 54.6% in total enrollment, marking the third consecutive year women dominated these three groups.^2^ With the growing number of women entering medicine, it is reasonable to conclude that fields traditionally male dominated, such as surgery, will see a similar change in demographic composition. For example, the representation of women in otolaryngology residency programs has increased, mirroring a broader trend in the medical field.^3^ Concurrent with the rise in female representation in this surgical subspecialty is the propensity to have children during residency training for both genders, spurring conversation on guidelines surrounding pregnancy and parental leave (PL).^4^^–^^6^

While institutional changes have been made to become more accommodating for female residents pregnant or hoping to have children during training, female physicians still face unique challenges and stigma when contemplating parenthood during their demanding training.^7^ Surgical residents especially possess lower perceptions of support in comparison to other specialties, although programs with female leadership did work to counter this perception.^8^ Contemporary discussion surrounding PL involves paternity leave as men have shown increased value in active parenting, which leads to continuous investment in caring for the child after paternity leave.^9^^,^^10^ Thus, it remains imperative for residency programs to recognize the roles of both men and women in becoming parents during training.

Such understanding is imperative to mitigate the negative associations and consequences of pregnancy during training. For example, studies demonstrate that pregnancy during surgical training may lead to negative peer evaluations, mother and child’s health, and even adverse obstetric complications.^7^^,^^11^^,^^12^ Furthermore, trainees report inadequate support, duration of leave, and dissatisfaction due to substantial income loss during PL.^11^^,^^13^ However, within the field of otolaryngology, there is little research on the attitudes of current male and female residents on the topic of PL, or resultant interventions.^14^^,^^15^ Therefore, this study examines the experiences and perspectives of otolaryngology residents affected by current parental leave (PL) practices, incorporating insights from both male and female resident physicians to assess the landscape of current institutional positions.

## Methods

Following Institutional Review Board approval from Medstar Georgetown University Hospital, a 43-item anonymous survey was generated using Qualtrics XM, a web-based survey tool. The survey was adapted from a study published in JAMA *Surgery* by Altieri et al. evaluating perceptions of surgical residents regarding PL in 2019.^1^

Our survey was distributed via email to all 125 Program Directors (PDs) of American College of Graduate Medical Education (ACGME)-accredited otolaryngology residency programs. We requested PDs to distribute the survey to all otolaryngology residents in their program, which yields 361 residents in total. For a population size of 361, at 95% confidence interval and margin of error of 10, we calculated a sample size of 77 respondents. Three separate emails were sent: an initial email, a follow-up email 30 days later, and a subsequent final email 60 days after the initial email was sent. A summary explanation of our research project was distributed together with the survey. Survey respondents were not compensated for their time.

The survey collected demographic information, information regarding PL policies at the respondents’ respective institutions, and queried respondents on perceived barriers to taking leave, perceived burden that PL places on a residency program, and perceived level of support from co-residents and faculty for those residents who took leave.

Survey results were collected from April 1, 2022 to June 30, 2022. XLSTAT Premium (Lumivero) was used to perform statistical analysis. Descriptive statistics were reported using the mean for continuous variables and frequencies and percentages for categorical variables. Summary statistics were used to describe characteristics of the survey responses. Using an inductive thematic analysis approach, free-text responses were reviewed to identify common themes and outliers among survey responses to determine the climate of perceptions on the indicated topics.

Of note, due to the anonymity of the survey respondents to establish confidentiality and ensure respondents felt capable to respond truthfully, we were unable to determine the breakdown of responses by program.

## Results

**Figure 1.**
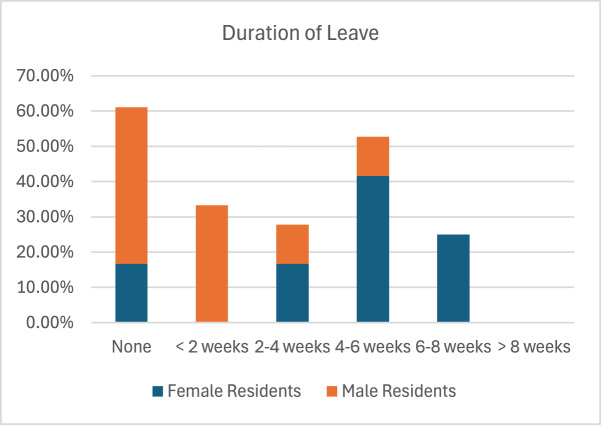
Duration of Leave.

Following the dissemination of our survey to all 125 (ACGME)-accredited otolaryngology residency programs, we had a response rate of 29% (105/361), which exceeded the necessary sample size of 77 responses. Most respondents were female (59, 57%), white (77, 74%), and married (80, 77%), with an average age of 30 years (range 25-35). The typical residency class size of survey respondents was 3.69 residents per class. Additional demographic data is described in Table 1a and Table 1b. Out of 105 respondents, 21 (20%) had biological children during residency, while 41 (39%) indicated that they were considering having children during training. Of those who had children during training, 15 (71.4%) took PL. Female residents typically took 4-6 weeks of leave, while male residents predominantly took none (Figure 1). No residents reported taking off more than 8 weeks of PL (Figure 1), and only 26 (25%) of respondents were aware of the PL policy of 8 weeks set by the American Board of Otolaryngology (ABO).

**Table 1a.** Resident respondent demographics

Demographic	N (%)
**Sex**	
Male	39 (42.9)
Female	52 (57.1)
**Race**	
White	67 (74.4)
Black	3 (3.3)
American Indian or Alaska Native	0 (0)
Asian	17 (18.9)
Pacific Islander	0 (0)
Other	3 (3.3)
**Ethnicity**	
Hispanic or Latino	4 (4.4)
Non-Hispanic or Latino	86 (95.6)
**Postgraduate Year**	
PGY−1	15 (16.9)
PGY−2	17 (19.1)
PGY−3	18 (20.2)
PGY−4	19 (21.4)
PGY−5	15 (16.9)
Fellow	5 (5.6)

**Table 1b.** Resident respondent program demographics

Region of Training Program	
Northeast	44 (49.4)
Southeast	14 (15.7)
Southwest	4 (4.5)
Midwest	21 (23.6)
West	6 (6.7)
**Size of Training Program**	
1 resident/year	3 (3.4)
2 residents/year	7 (7.9)
3 residents/year	35 (39.3)
4 residents/year	22 (24.7)
5 residents/year	14 (15.7)
6 residents/year	8 (9.0)
**Relationship Status**	
Married or domestic partnership	69 (77.5)
Divorced, separated, or widowed	0 (0)
Single	20 (22.5)
**Occupation of Spouse or Partner**	
Non-physician	50 (73.5)
Attending physician	3 (4.4)
Resident physician	15 (22.1)

**Figure 2.**
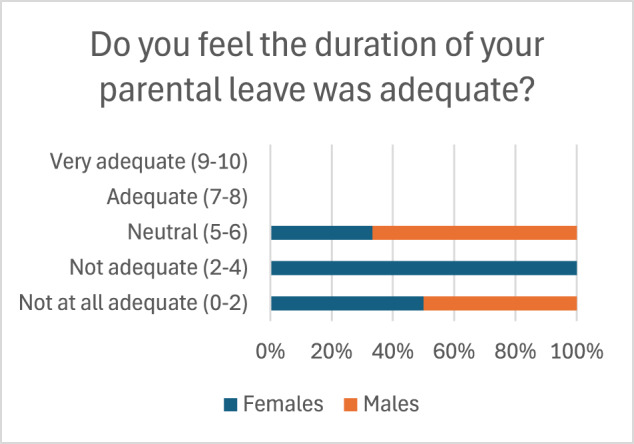
Perception of Duration of Leave.

**Figure 3.**
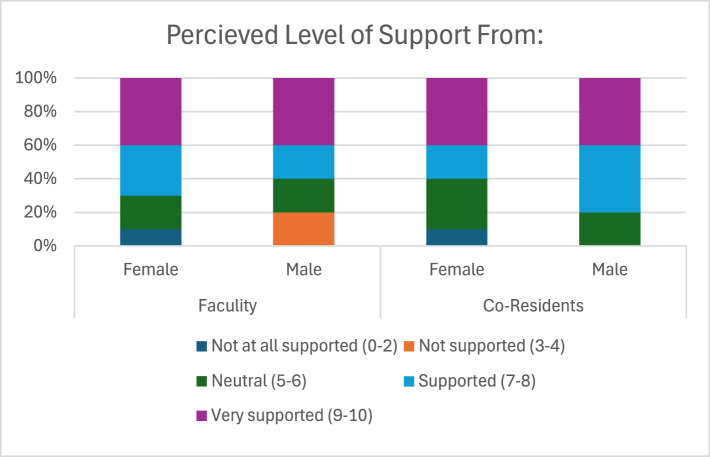
Perception of Support from Program.

**Figure 4.**
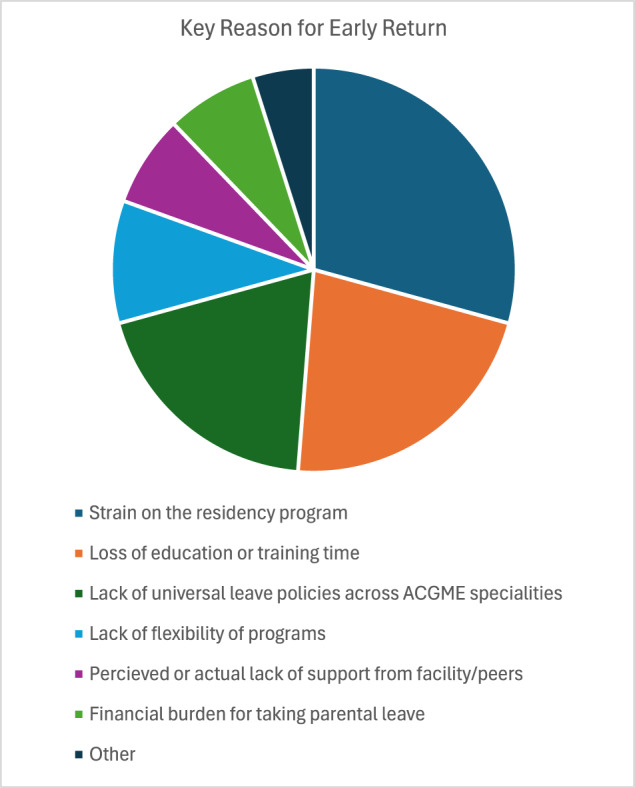
Key Reason for Early Return.

Regarding satisfaction in PL policies, 90% of female residents and 100% of male residents felt their leave duration was inadequate (Figure 2). Most respondents who took leave commented that they felt supported in doing so by their co-residents and faculty (Figure 3). Female residents reported lower perceived levels of support from co-residents than male residents (60% vs 80%, respectively), but higher perceived levels of support from faculty (70% vs 60%, respectively) (Figure 3). Figure 4 displays respondents’ concerns with taking PL leave during residency, where fear of placing strain on the program was the most common concern (29.3%), followed by loss of education or training time (22%), and lack of universal leave policies across ACGME specialties (19.5%).

Concerning lactation support in the post-partum period, 91.6% breastfed or pumped upon return to work, but 60% felt they lacked sufficient time, and 63.6% had no dedicated space for pumping. Specifically, when asked which spaces female residents used to pump after returning to residency training, free-response answers included: “my car,” “the locker room shower,” “in the resident room in front of co-residents,” and “the shower stall in our bathroom.” Only one survey respondent indicated that she was provided with a dedicated lactation space for pumping.

## Discussion

Residency programs are hindered from providing trainees dedicated time off for personal obligations by their obligation to ensure continuous delivery of quality patient care, optimization of surgical training, and avoid violation of work hour parameters allotted to other residents under the ACGME regulations. Our study demonstrates a pioneering effort to elucidate experiences and perspectives of otolaryngology residents affected by current parental leave (PL) practices. Despite the existence of current guidelines attempting to alleviate parent’s needs pre- and post-partum, the unanimous dissatisfaction with current PL policies among the otolaryngology residents surveyed demands a re-evaluation of such institutional PL regulations.

Current ABO policies guarantee a maximum of 6 weeks of paid consecutive parental, caregiver, or medical leave (8 weeks including vacation time).^16^ Our data indicates that 79 (75%) of respondents were unaware of the ABO PL policy, which likely contributed to the shorter durations in PL taken by both female and male residents (4-6 weeks and 0 weeks on average, respectively). While the reasons behind inadequate utilization of parental leave can only be hypothesized based on these subjective survey responses—such as a lack of knowledge of ACGME policy, personal resident choices, fear of loss of wages, or implicit or explicit pressures of the residency program—a potential violation of ACGME policy could be inferred. If residents are unaware of their rights or feel pressured not to take leave, programs might not be complying with ACGME’s mandate for six weeks of paid leave. Ensuring awareness and enforcement of this policy is crucial to prevent such violations.

This limited parental leave policy in the U.S. stands in stark contrast to policies in other countries. In Canada, paid parental leave is federally protected, whereas the U.S. lacks a national policy guaranteeing paid leave for medical trainees, offering only unpaid, job-protected leave through the Family and Medical Leave Act (FMLA). Many other countries, such as Australia and those in the European Union, have national leave policies that apply to their medical trainees. In the UK, for example, the majority of residents take 6-12 months of parental leave, underscoring the disparity in support structures for physicians in training. Meanwhile, in the U.S., the ACGME and the American Board of Medical Specialties (ABMS) mandate a maximum of six weeks of parental leave, inclusive of vacation time—far less than what is available in many other countries.

Thus, we recommend residency programs to sufficiently address this discrepancy to ensure residents can and do take advantage of this policy. At the same time however, even if residents do learn about the PL policy, they still may take a shorter duration of PL due to fear of placing strain on the program, loss of education or training time, perceived lack of support, and financial burden, among others (Figure 4). Moreover, given that many otolaryngology residency programs require that vacation is taken in week-long increments, it may also be the case that vacation time is difficult to preserve for PL alone.

Additionally, the PL policy often intersects with the Catastrophic Leave Policy available in certain hospital systems. This latter policy allows residents to take an extended leave of up to eight to ten weeks for significant personal or family medical emergencies without extending their training period.^17^ Residents who use the eight-week period for parental leave are ineligible for this additional policy, potentially leaving them without further leave for unforeseen events such as illness or family deaths. Investigating the perceptions and impacts of this policy could provide valuable insights for future studies.

Given that 90% of female residents and 100% of male residents felt that PL was inadequate, residents inadvertently weighed the risks and benefits of pregnancy and having children during training. Deciding to wait until after training incurs biological penalties, as evidenced by higher rates of infertility in female surgeons (32% vs 10.2%) compared to the general population, especially female otolaryngologists.^18^ At the same time, those who decide to have children during training face higher rates of obstetric complications including but not limited to preterm labor and pre-eclampsia compared to non-medical wives of male residents.^19^ Thus, programs should not only consider such risks when deciding on policies, but incorporate the perspectives, preferences, and expectations of their residents to inform PL policies.

Our survey found that many residents are worried about the strain their absence could cause for their co-residents when taking leave. Unlike larger residency programs such as General Surgery, Pediatrics, and Internal Medicine, where cohorts can exceed ten residents per year, the average number of residents per post-graduate year class among those surveyed was 3.69.^20^ Consequently, a single resident’s absence could significantly burden the remaining residents. Despite this concern, most residents don’t believe that parental leave places undue strain on the residency program. This suggests a subjective disconnect between residents’ concern for their co-residents’ burden and their actual perspectives, which warrants an open discussion within programs to clear up misunderstandings and devise more efficient systems to mitigate real and perceived burden.

Other notable concerns include support for postpartum lactation practices as female residents return to work. Our survey results demonstrated most women reported not being given adequate time to pump at work or not being provided with a dedicated space to pump and store breastmilk. Such concerns have been addressed through the Providing Urgent Maternal Protections for Nursing Mothers (“PUMP”) Act.^21^ Under the Fair Labor Standards Act and U.S. Department of Labor, employers must provide “…a reasonable amount of break time and a space to express milk as frequently as needed by the nursing mothers, for up to one year following the birth of the employee’s child.”^22^ Furthermore, the space provided “…cannot be a bathroom and must be shielded from view and free of intrusion by coworkers or the public.”^22^ However, as can be deduced from our survey, such regulations are frequently neglected for female otolaryngologists, as they had to find semi-private areas on their own volition, unsupported by the institutions. The PUMP Act’s detailed requirements for time and space for nursing mothers upon returning to work show that these challenges aren’t exclusive to surgical residents. The need for such accommodations doesn’t imply weakness or inability to complete rigorous surgical training; it simply recognizes that nursing adds to the physical demands of training, necessitating basic support. Residency programs must work expeditiously to correct deficiencies in lactation support to alleviate the undue burden, stress, and resources female residents must face during their training in the post-partum period.

This study has several limitations. Due to the small number of otolaryngology residents nationwide (361 spots filled), our sample size was limited to 105 participants. Response bias may exist as residents with personal experiences related to pregnancy and parental leave may have been more likely to participate. The high proportion of female respondents (57%) compared to the overall otolaryngology resident population (34.7% female) suggests potential bias. Additionally, without a database tracking residents’ pregnancy or parenthood status, the survey’s effectiveness in capturing the opinions of all affected residents is uncertain. Lastly, the study did not explore assistance opportunities like subsidized childcare, which could affect financial strain and warrant future investigation.

- What is Known:
  - Medical schools have an increasing percentage of female enrollees, who are matriculated into more male-dominated surgical fields – i.e. Otolaryngology
  - Parental leave policies are critical as more residents, both male and female, consider parenthood during training
  - PL policies often lack standardization, and residents report limited support and high rates of dissatisfaction with leave policies across surgical specialties
- What This Paper Adds:
  - Provides quantitative and qualitative data on Otolaryngology residents’ views regarding current PL practices
  - Reveals only 25% of residents are aware of the American Board of Otolaryngology’s PL policy of eight weeks
  - Identifies key barriers to taking PL, such as fear of program strain, inadequate leave duration, and financial burden
  - Highlights inadequate postpartum lactation support, with 60% of respondents reporting insufficient time and space for breastfeeding or pumping
  - Suggests need for re-evaluation of PL policies to address residents’ concerns, improve awareness, and ensure adequate support across residency programs

## Conclusion

In conclusion, our study sheds light on the experiences and perspectives of otolaryngology residents regarding parental leave practices, incorporating insights from both male and female resident physicians. Despite efforts to accommodate residents with parental obligations, dissatisfaction with current policies persists among surveyed residents, highlighting the need for a re-evaluation of institutional regulations. Lack of awareness of existing policies, fear of program strain, and concerns about support and financial burden contribute to shorter leave durations and stress among residents. Furthermore, inadequate lactation support underscores ongoing challenges faced by female residents returning to work postpartum. The findings emphasize the importance of addressing these issues to ensure equitable support for all residents regardless of gender. While our study has limitations, it underscores the urgency for further research and action to enhance parental leave policies and support mechanisms within residency programs. Efforts to address these challenges are essential to fostering a supportive and inclusive environment for all residents pursuing parenthood during their training.

## References

[1] Altieri MS, Salles A, Bevilacqua LA, et al. Perceptions of Surgery Residents About Parental Leave During Training. JAMA Surg 2019;154:952–958 31389989 10.1001/jamasurg.2019.2985 PMC6686777

[2] Henry TA. Medical student diversity sees uptick—for now. Published December 21, 2023. https://www.ama-assn.org/education/medical-school-diversity/medical-student-diversity-sees-uptick-now#:∼:text=Women%20accounted%20for%2056.6%25%20of,majority%20of%20these%20three%20groups [20 March 2024]

[3] Active Physicians by Sex and Specialty, 2019. Physician Specialty Report Data. https://www.aamc.org/data-reports/workforce/data/active-physicians-sex-and-specialty-2019 [20 March 2024]

[4] Holliday EB, Ahmed AA, Jagsi R, et al. Pregnancy and Parenthood in Radiation Oncology, Views and Experiences Survey (PROVES): Results of a Blinded Prospective Trainee Parenting and Career Development Assessment. Int J Radiat Oncol Biol Phys 2015;92:516–524 25892584 10.1016/j.ijrobp.2015.02.024

[5] Smith C, Galante JM, Pierce JL, Scherer LA. The surgical residency baby boom: changing patterns of childbearing during residency over a 30-year span. J Grad Med Educ 2013;5:625–629 24455012 10.4300/JGME-D-12-00334.1 PMC3886462

[6] Turner PL, Lumpkins K, Gabre J, Lin MJ, Liu X, Terrin M. Pregnancy among women surgeons: trends over time. Arch Surg Chic Ill 1960 2012;147:474–479 10.1001/archsurg.2011.1693 22351877

[7] Rangel EL, Castillo-Angeles M, Changala M, Haider AH, Doherty GM, Smink DS. Perspectives of pregnancy and motherhood among general surgery residents: A qualitative analysis. Am J Surg 2018;216:754–759 30072028 10.1016/j.amjsurg.2018.07.036

[8] Mundschenk MB, Krauss EM, Poppler LH, et al. Resident perceptions on pregnancy during training: 2008 to 2015. Am J Surg 2016;212:649–659 27575602 10.1016/j.amjsurg.2016.06.018

[9] Huerta MC, Adema W, Baxter J, et al. Fathers’ Leave and Fathers’ Involvement: Evidence from Four OECD Countries. Eur J Soc Secur 2014;16:308–346 28479865 10.1177/138826271401600403 PMC5415087

[10] Jones J, Mosher WD. Fathers’ involvement with their children: United States, 2006-2010. Natl Health Stat Rep 2013;71:1–21 24467852

[11] Castillo-Angeles M, Atkinson RB, Easter SR, et al. Pregnancy During Surgical Training: Are Residency Programs Truly Supporting Their Trainees? J Surg Educ 2022;79:e92–e102 35842402 10.1016/j.jsurg.2022.06.011

[12] Todd AR, Cawthorn TR, Temple-Oberle C. Pregnancy and Parenthood Remain Challenging During Surgical Residency: A Systematic Review. Acad Med J Assoc Am Med Coll 2020;95:1607–1615 10.1097/ACM.0000000000003351 32271231

[13] Scully RE, Davids JS, Melnitchouk N. Impact of Procedural Specialty on Maternity Leave and Career Satisfaction Among Female Physicians. Ann Surg 2017;266:210–217 28272103 10.1097/SLA.0000000000002196

[14] Champaloux EP, Acosta AS, Gray ST, Meyer TK, Bergmark RW. Otolaryngology residents’ experiences of pregnancy and return to work: A multisite qualitative study. Laryngoscope Investig Otolaryngol 2022;7:1322–1328 10.1002/lio2.878 PMC9575055 36258851

[15] Cole S, Arnold M, Sanderson A, Cupp C. Pregnancy during otolaryngology residency: experience and recommendations. Am Surg 2009;75:411–415 19445293

[16] Parental, Caregiver and Medical Leave During Training. Residency & Training, Policy & Governance. Published March 1, 2021. https://www.abohns.org/parental-caregiver-and-medical-leave-during-training [20 March 2024]

[17] 2116 - Catastrophic Leave. Human Resources Manual. https://hrmanual.calhr.ca.gov/Home/ManualItem/1/2116 [17 June 2024]

[18] Phillips EA, Nimeh T, Braga J, Lerner LB. Does a surgical career affect a woman’s childbearing and fertility? A report on pregnancy and fertility trends among female surgeons. J Am Coll Surg 2014;219:944–950 25260684 10.1016/j.jamcollsurg.2014.07.936

[19] Klebanoff MA, Shiono PH, Rhoads GG. Outcomes of pregnancy in a national sample of resident physicians. N Engl J Med 1990;323:1040–1045 2215563 10.1056/NEJM199010113231506

[20] Table B3. Number of Active Residents, by Type of Medical School, GME Specialty, and Gender. Report on Residents. Accessed March 20, 2024. https://www.aamc.org/data-reports/students-residents/data/report-residents/2023/table-b3-number-active-residents-type-medical-school-gme-specialty-and-gender [20 March 2024]

[21] The Providing Urgent Maternal Protections (PUMP) For Nursing Mothers Act. United States Breastfeeding Committee. https://www.usbreastfeeding.org/pump-act.html [20 March 2024]

[22] Fact Sheet #73: FLSA Protections for Employees to Pump Breast Milk at Work. Wage and Hour Division. Published January 1, 2023. https://www.dol.gov/agencies/whd/fact-sheets/73-flsa-break-time-nursing-mothers [20 March 2024]

